# A Synergistic Effect of Surfactant and ZrO_2_ Underlayer on Photocurrent Enhancement and Cathodic Shift of Nanoporous Fe_2_O_3_ Photoanode

**DOI:** 10.1038/srep32436

**Published:** 2016-08-31

**Authors:** Pravin S. Shinde, Su Yong Lee, Sun Hee Choi, Hyun Hwi Lee, Jungho Ryu, Jum Suk Jang

**Affiliations:** 1 Division of Biotechnology, Division of Biotechnology, Safety, Environment and Life Science Institute, College of Environmental and Bioresource Sciences, Chonbuk National University, Iksan 570-752, Republic of Korea; 2 Pohang Accelerator Laboratory, Pohang University of Science and Technology (POSTECH), Pohang 790-784, Republic of Korea; 3 Mineral Resources Research Division, Korea Institute of Geoscience and Mineral Resources (KIGAM), Daejeon 305-350, Republic of Korea

## Abstract

Augmenting the donor density and nanostructure engineering are the crucial points to improve solar water oxidation performance of hematite (α-Fe_2_O_3_). This work addresses the sluggish water oxidation reaction associated with hematite photoanode by tweaking its internal porosity. The porous hematite photoanodes are fabricated by a novel synthetic strategy via pulse reverse electrodeposition (PRED) method that involves incorporation of a cationic CTAB surfactant in a sulfate electrolyte and spin-coated ZrO_2_ underlayer (UL) on FTO. CTAB is found to be beneficial in promoting the film growth rate during PRED. Incorporation of Zr^4+^ ions from ZrO_2_ UL and Sn^4+^ ions from FTO into the Fe_2_O_3_ lattice via solid-state diffusion reaction during pertinent annihilation of surfactant molecules at 800 °C produced internally porous hematite films with improved carrier concentration. The porous hematite demonstrated a sustained photocurrent enhancement and a significant cathodic shift of 130 mV relative to the planar hematite under standard illumination conditions (AM 1.5G) in 1 M NaOH electrolyte. The absorption, electrochemical impedance spectroscopy and Mott-Schottky analyses revealed that the ZrO_2_ UL and CTAB not only increased the carrier density and light harvesting but also accelerated the surface oxidation reaction kinetics, synergistically boosting the performance of internally porous hematite photoanodes.

Hematite (α-Fe_2_O_3_) has sustained its utilization in solar water splitting due to highest theoretical promise in conversion efficiency (12.9%) among all other metal oxide semiconductors^1^. Besides, it has a suitable band gap (*E*_g_ = 2.1 eV) within the visible light spectrum regime and possesses good electrochemical stability^2^. However, intrinsically poor conductivity of hematite is one of the major impeding factors, which limits its practical usage in solar energy conversion. Other factors include a low optical absorption coefficient (due to a short light penetration depth of ~118 nm)^3^ in spite of a wide visible absorption range (300–500 nm)^4^ and a high electron-hole recombination rate (due to a short hole diffusion length of ~2–4, 20 nm)^5^,^6^. Thus, the Fe_2_O_3_ (α is dropped hereafter) thickness for water splitting is limited to a few hundred nanometers due to its poor absorptivity and short hole diffusion length. Consequently, Fe_2_O_3_ suffers sluggish water oxidation kinetics due to intrinsically poor conductivity, poor absorptivity and a high electron-hole recombination rate. These demerits of Fe_2_O_3_ can potentially be overcome through a variety of strategies^7^, including doping to enrich conductivity or donor density^8^,^9^,^10^,^11^, nano-texturing or engineering the nano-architectures to improve light trapping^12^, and use of co-catalysts^13^,^14^. Over the past few years, doping and nanostructuring have shown overwhelming effects on both the photocurrent as well as the onset potential of Fe_2_O_3_. The heteroatom doping coupled with an appropriate nanostructuring of hematite has served as an effective approach for improving the photoelectrochemical (PEC) performance of hematite by increasing carrier concentration and/or charge mobility^8^,^9^,^12^,^15^,^16^. Several strategies such as *in-situ* reaction, surface-treatment, and the use of overlayers and/or underlayers, etc. are employed to thermally activate hematite via diffusion doping. Among the various dopants (Ti, Sn, Si, Ge, Zr, etc.), the Ti^4+^ and Sn^4+^ ions have been predominantly used in mono- or co-doped configurations^9^. However, from quantum mechanical point of view, the electron transport in hematite with Zr doping is superior compared to Ti^17^, because Zr^4+^ does not trap electrons due to a low ionization potential and stability and is experimentally proved^18^,^19^. Recently, thin metal oxide underlayers (layers coated on FTO substrate) have shown dramatic improvement in the PEC performance of hematite^20^,^21^. The underlayer (UL) not only improved interfacial properties but also enriched the donor concentration of hematite via diffusion doping upon high-temperature (HT) annealing. Thus far, the UL effects have only been studied for ultrathin compact hematite films (<50 nm), using TiO_2_, SiO_x_, Nb_2_O_5_, and Ga_2_O_3_ unerlayers^22^. In fact, such effect can be applied to relatively thick nanostructures that could absorb more light by modulating the optical and charge transport path lengths. This notion can be met by tailoring the porosity of hematite nanostructures. Higher porosity would improve the photocurrents due to an increase in electrolyte/hematite interfaces within the depletion width of the absorption sites. For instance, the use of mesoporous materials has been a common practice to improve performance in dye sensitized solar cells^23^. The porous nanostructures can be obtained through the use of templates or surfactants. The pulse reverse electrodeposition (PRED) method offers one of the simplest, cost-effective, and scalable ways to achieve porous nanostructures. It can be used to fabricate thin films with nano-sized dimensions in a homogeneous manner with great control over film thickness^24^. Incorporating surfactant into the electrolyte is a straight forward method to control nanostructured growth, porosity, and the crystallinity of electrodeposited materials^25^. Surfactants are previously employed as templates to obtain Fe_2_O_3_ nanoparticles, but their utilization for fabrication of iron/iron oxide films via any electrochemical methods has not yet been performed. Among the various surfactants, cetyltrimethylammonium bromide (CTAB) is an amine based cationic surfactant (See Fig. S1 in Supplementary Information) widely used as a stabilizer and structure-directing agent to control nucleation and growth of the crystallites^26^. Herein, we report a new synthetic approach that involves fabrication of internally porous hematite thin films on ZrO_2_ UL-coated FTO via a facile PRED method by employing a cationic CTAB surfactant in the sulfate electrolyte and the implementation of resulting photoanodes for efficient PEC water splitting. The effects of CTAB and ZrO_2_ UL on the morphological, crystalline orientation, conductivity, and PEC properties of hematite are discussed. For the first time, a ZrO_2_ UL on FTO is employed to fabricate Fe_2_O_3_ films. This is also the first attempt to fabricate photoactive intra-porous Fe_2_O_3_ photoanode using PRED with the help of CTAB and ZrO_2_ UL.

## Results and Discussion

**Figure 1:**
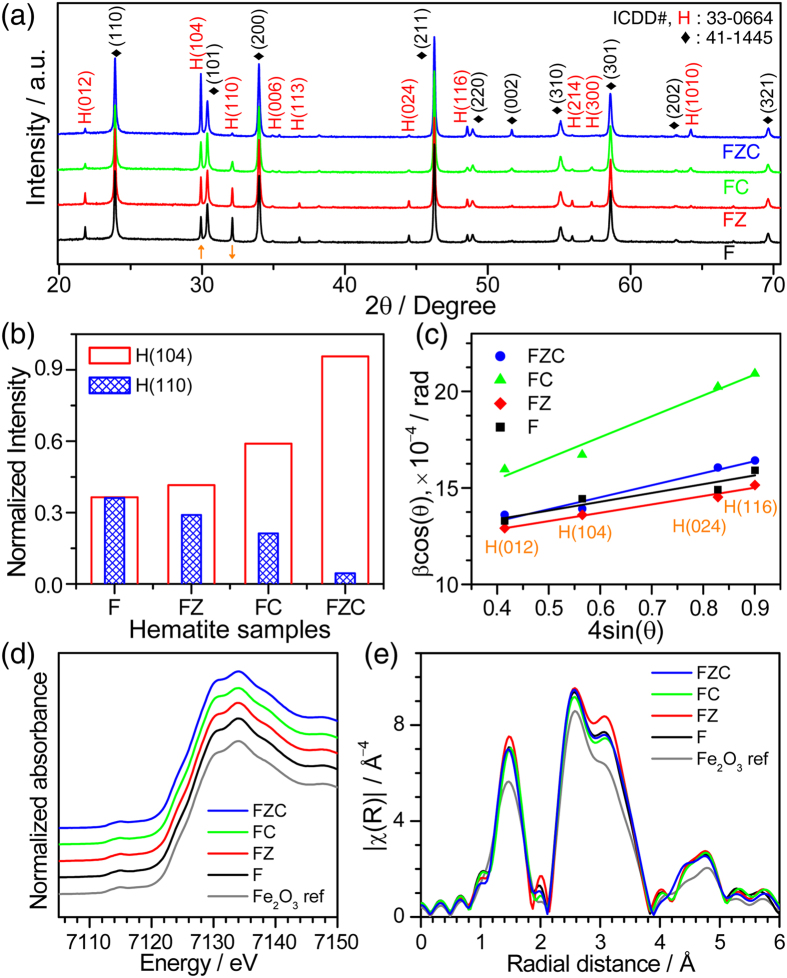
(**a**) The conventional θ–2θ XRD scans (λ = 0.1393 nm) of Fe_2_O_3_ photoanodes fabricated using UL and surfactant, (**b**) Changes in normalized diffraction intensity of hematite planes, and (**c**) Williamson-Hall plot. (**d**) Fe K-edge XANES spectra, and (**e**) *k*^3^-weighted Fourier transforms of Fe K-edge EXAFS functions for different Fe_2_O_3_ photoanodes.

The Fe_2_O_3_ films prepared by PRED without, with UL alone, with CTAB alone, and with both UL and CTAB are denoted as F, FZ, FC, and FZC, respectively (see Materials synthesis section). The structural studies of iron oxide films are performed using synchrotron XRD patterns recorded in the diffraction angle (2θ) range of 20–70°. As shown in Fig. 1a, all the peaks are indexed to the hematite phase (α-Fe_2_O_3_, denoted as ‘H’, JCPDS 33–0664) and substrate FTO phase (SnO_2_, denoted as ‘♦’, JCPDS 41–1445). Significant changes according to the synthesis conditions are observed in the peak intensities of major hematite (104) and (110) Bragg reflections. To clarify the intensity variation, the H(104), H(110), and ♦(101) peak profiles are first fitted with the Pearson-VII function and the integrated intensity of each peak is obtained. Then, the integrated intensities of the H(104) and H(110) peaks normalized by the substrate F(101) peak are plotted for all the sample conditions as shown in Fig. 1b. The sample F fabricated without UL and surfactant showed almost similar contribution from H(110) and H(104) planes. For FZ sample, the H(104) intensity increased with slight decrease in H(110) intensity. This variation in the integrated intensity is greatly enhanced through the introduction of CTAB surfactant alone. Furthermore, the intensity of H(104) is dramatically increased by 162%, whereas that of H(110) is reduced by 88% for FZC condition when both a ZrO_2_ UL and CTAB are employed. This suggests that CTAB surfactant promotes or suppresses the formation of H(104) or H(110) hematite planes, respectively. Thus, the surfactant markedly affects the crystalline orientation of hematite. In order to evaluate the structural properties such as crystallite size and micro strain, Williamson-Hall analysis is applied to the line profiles converted from the 2D XRD patterns rather than using θ–2θ line profiles. Since the converted profile is obtained by integrating a ring pattern along the circular arc, the 2D XRD pattern is advantageous toward analyzing weak Bragg diffraction peaks. The average crystallite size (*D*) and micro-strain (*ε*_μ_) values of a particular phase are estimated using equation (1)^27^:


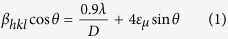


where β is the full width at half maximum (FWHM) of the peak from the (*hkl*) plane, θ is the Bragg angle. The FWHM and θ values for the hematite peaks are obtained by fitting the line profiles (using Pearson−VII function) acquired from 2D XRD scans (Fig. S2). Figure 1c shows the Williamson-Hall plots (βcos θ versus 4sin θ) for all the Fe_2_O_3_ photoanodes. Table S1 depicts the *D* and *ε*_μ_ values evaluated from the intercept and the slope of each fitted line, respectively. The average crystallite size remained 60 nm regardless of synthesis condition. However, it is worth mentioning that the micro-strain is distinctly increased upon the use of surfactant (FC sample) by 135% compared to the sample F. The FZC sample showed moderate *ε*_μ_ value upon use of ZrO_2_ UL, which indicates that Zr^4+^ ion incorporation relaxes the strain in hematite to some extent. While XRD characterizes the long range order and structure of the whole compound, XAFS is employed to examine the local structures around a specific element with a low concentration-sensitivity. Fe K-edge X-ray absorption near edge (XANES) spectra for the samples are perfectly identical to that of α-Fe_2_O_3_ reference, where the pre-edge peak denoting a quadrupole transition from the 1*s* core electron to the unoccupied 3*d* level is observed at 7115 eV and the electric dipole transition feature to an empty 4p level is also same (Fig. 1d). This proved that the electronic structure of the photoanodes is not perturbed by the UL or the surfactant. Figure 1e displays the Fourier transforms of the extended X-ray absorption structure (EXAFS) functions for the Fe K-edge of the photoanodes. The spectra exhibit two peaks; the first peak at 0.8–1.9 Å due to interaction between a central Fe atom and the nearest O neighbors and the other peak at 2.1–3.9 Å due to complicated Fe–Fe and Fe–O scatterings with longer bond distances. A slight increase in peak intensities is a usual indication of enhanced bond ordering for the films on the substrate. However, the surfactant and the UL unaffected the structures of the Fe_2_O_3_ photoanodes in an angstrom order.

**Figure 2:**
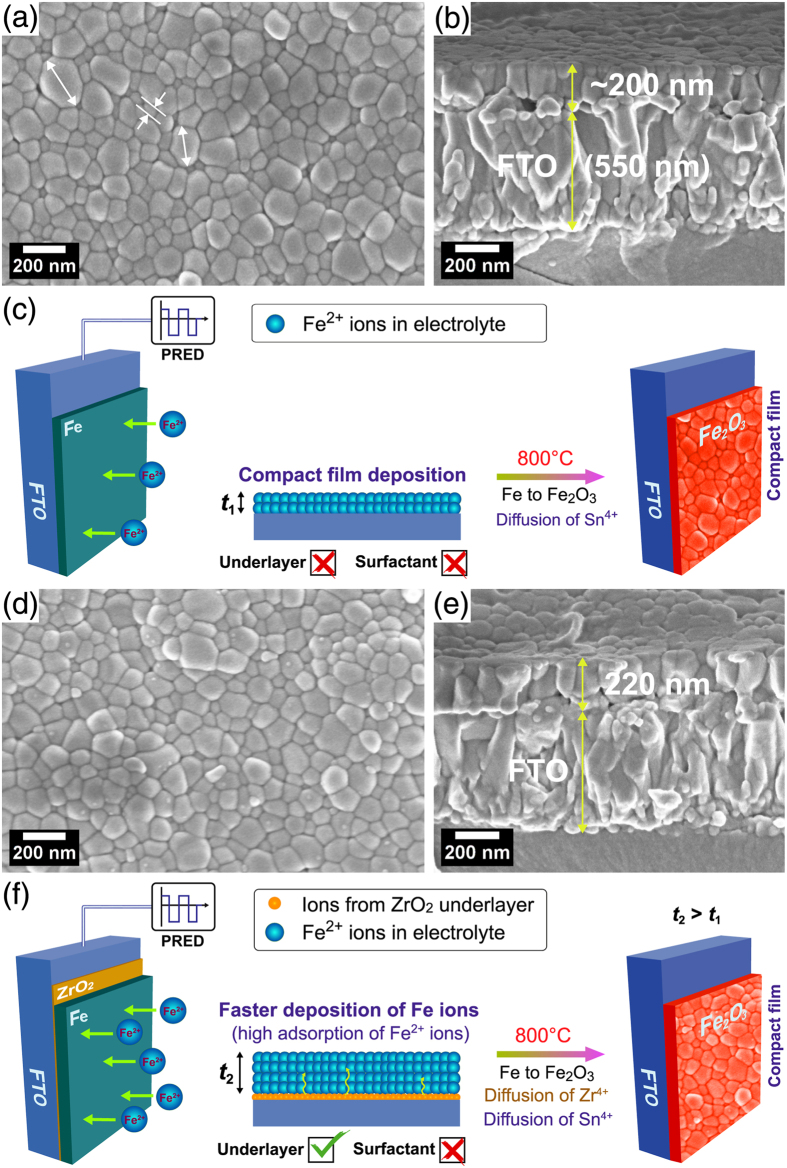
Surface and cross-sectional FESEM images of Fe_2_O_3_ photoanodes prepared with (**a,b**) F and (**d,e**) FZ conditions. Figure (**c**,**f**) shows the schematic of film formation on FTO for F and FZ conditions, respectively.

**Figure 3:**
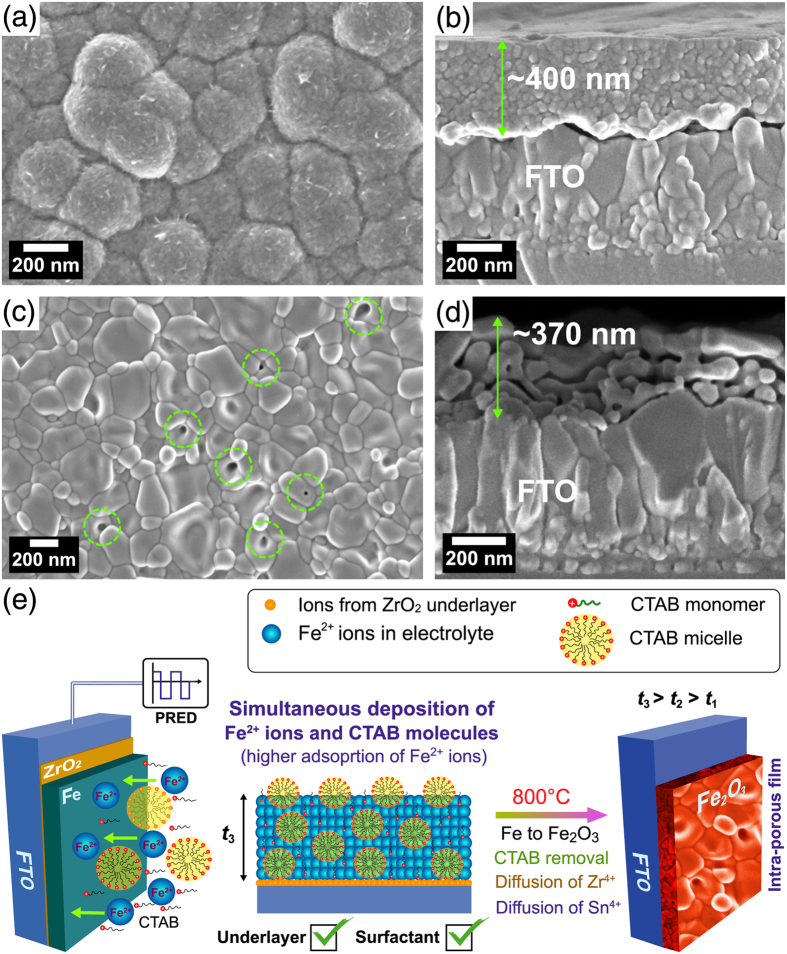
Surface and cross-sectional FESEM images of (**a,b**) as-grown Fe and (**c,d**) annealed Fe_2_O_3_ films, and (**e**) schematic of film formation on FTO for FZC condition.

Figure 2a,b show the surface and cross-sectional FESEM images of Fe_2_O_3_ film (~200 nm) fabricated in the absence of ZrO_2_ UL and CTAB surfactant revealing compact nano-structured morphology with good particle inter-connectivity. The FESEM image distinctly shows different grain size distributions, exhibiting larger and smaller sized grains of 170 ± 25 and 60 ± 25 nm, respectively. Figure 2c shows the schematic of a compact Fe_2_O_3_ film (thickness *t*_1_) formation from Fe film. HT-annealing of Fe film at 800 °C causes the partial release of Sn^4+^ ions from deformed FTO, which are diffused and doped unintentionally into the crystallizing Fe_2_O_3_ lattice. With the use of ZrO_2_ UL alone, the large and small grain distribution is changed to 150 ± 30 nm and 80 ± 20 nm, respectively (Fig. 2d,e). The thickness of the ZrO_2_ UL on glass before HT-annealing estimated from spectroscopic ellipsometry analysis (Fig. S3) is 38±0.2 nm. The coverage of ZrO_2_ UL on FTO may however be different because FTO has high surface roughness unlike glass. The film morphology of Fe_2_O_3_ prepared using ZrO_2_ UL is still granular and compact with a slightly increased film thickness of 220 nm. This is because the adsorption rate of Fe^2+^ ions on amorphous ZrO_2_–coated FTO during PRED might have increased leading to higher mass of Fe film (thickness *t*_2_ > *t*_1_). Upon HT-annealing, both Sn^4+^ and Zr^4+^ ions are concurrently diffused into the crystallizing Fe_2_O_3_ lattice. However, the solid state diffusion of Zr^4+^ ions from UL is lower since their concentration as well as the diffusion rate is relatively smaller than that of Sn^4+^ ions (Fig. 2f). When both the ZrO_2_ UL and CTAB surfactant are employed, altogether different morphology is observed. To explain this it is necessary to study the morphology of as-grown Fe film prepared using FTC condition. Figure 3a,b show the FESEM images of as-grown Fe film revealing the spherical clusters (250 ± 40 nm) comprising Fe grains and nanorod-like spikes with relatively higher film thickness of 400 nm. The surface morphology of annealed Fe_2_O_3_ film reveals further increment in larger and smaller grain sizes to 245 ± 40 and 95 ± 20 nm, respectively, forming a flat film with several pores on the surface (Fig. 3c,d). The surface morphologies of all the hematite samples fabricated by HT-annealing in the present study show two grain distributions. Microstructure evolution of all the hematite photoanodes follows a well-known phase field model^28^, which can be explained by Zener pinning phenomenon. Zener pinning is the influence of a dispersion of fine particles on the movement of low- and high angle grain boundaries through a polycrystalline material. Small particles act to prevent the motion of such boundaries by exerting a pinning pressure, which counteracts the driving force pushing the boundaries. In other words, the grain growth phenomena can be separated into continuous and discontinuous mechanisms. In the former case, the microstructure evolves from one state to another and grains become larger in a uniform manner. In the latter case, the changes occur heterogeneously and specific transformed and untransformed regions may be identified. Abnormal or discontinuous grain growth, also referred to as exaggerated or secondary recrystallization grain growth, is a grain growth phenomenon through which certain energetically favorable grains grow rapidly in a matrix of finer grains resulting in a bimodal grain size distribution^29^. The discontinuous grain growth is characterized by a subset of grains growing at a high rate and at the expense of their neighbors and tends to result in a microstructure dominated by a few very large grains. This occurs when the subset of grains possess a high grain boundary energy, locally high grain boundary mobility, favorable texture or lower local second-phase particle density (Sn and Zr particles in the present work). The cross-sectional FESEM image of FZC sample reveals an internally porous network with a film thickness of 370 nm. The hematite layers of 70–80 nm are spatially connected to each other by a distance of ~10–20 nm. The pores seen on the surface appears to have their connections into to the porous network. In this case, the film formation mechanism is largely influenced by CTAB surfactant (Fig. 3e). CTAB is an organic molecule consisting of positively charged nitrogen and negatively charged bromine ion connected by a chain of several methyl groups. CTAB finds potential applications in nano-technology due to its strong adsorbing ability of nano particles. Here, CTAB plays a vital role in controlling the nano-architecture design under the template effect. Generally, surfactant in aqueous solution at different concentrations can form into different shapes in aqueous solution^30^. The aggregation process of surfactant molecules may lead to the formation of different mesoscopic aggregates, including: micelles, vesicles, bilayers, and mesophases with various geometries^31^. The precise structures of these aggregates depends on the type and concentration of surfactant, temperature, and presence of electrolytes or organic additives, among others^32^. The concentration at which micelle formation starts is known as critical micelle concentration (CMC). The CTAB concentration in our work is notably above the CMC value (typically < 1 mM for CTAB). The micelles exhibit spherical shapes at concentrations not far from CMC^31^. The surfactant ions (including CTAB monomers and micelles) adsorb as counter ions at the solvated Fe^2+^-liquid interface by electrostatic attraction forming a loose complex. Under the action of potential during PRED, the adsorption density of surfactant is sufficiently high causing higher deposition rate of surfactant-bounded Fe^2+^ ions toward the ZrO_2_/FTO substrate. Thus, the ZrO_2_ UL helps in adsorption of Fe^2+^ ions and the CTAB micelles further accelerate the deposition rate of Fe^2+^ ions, leading to significantly higher deposition of Fe mass (thickness *t*_3_ > *t*_2_ > *t*_1_). Upon HT-annealing, the surfactant molecules are gradually annihilated, producing voids in the crystallizing Fe_2_O_3_ lattice. Meanwhile, the Zr^4+^ from ZrO_2_ UL and Sn^4+^ ions from FTO are also migrated into the crystallizing Fe_2_O_3_ lattice via solid state diffusion. During onset of pore formation, the crystallized hematite layers collapse and re-crystallize again while interacting with the diffused cations. The top surface of Fe_2_O_3_ is settled to form a flat layer after re-crystallizations at high temperature, forming few pores that are connected to porous network. Thus, internally porous nanostructured hematite photoanode with higher conductivity is achieved. Such internally-porous network helps in photon-trapping via multiple light-scattering to yield large number of photogenerated electron-hole pairs and also engage maximum adsorption of electrolyte via the percolation pathways through voids on the surface. As generally accepted, Fe_2_O_3_ suffers from short hole diffusion length in the bulk and swift charge recombination on the surface, which adversely affects the PEC response of Fe_2_O_3_ as a photoanode^33^. However, our porous FZC film consists of 50–60 nm hematite layers which overcome the short diffusion length problem of photogenerated carriers to some extent. The Fe_2_O_3_ film prepared only using CTAB surfactant (FC condition) reveals a compact morphology without the surface pores (Fig. S4). However, the film growth is patchy resulting in non-uniform hematite coverage upon annealing. This also confirms the importance of ZrO_2_ UL for assisting the uniform film deposition as well as for improving the interface of Fe_2_O_3_ and FTO.

**Figure 4:**
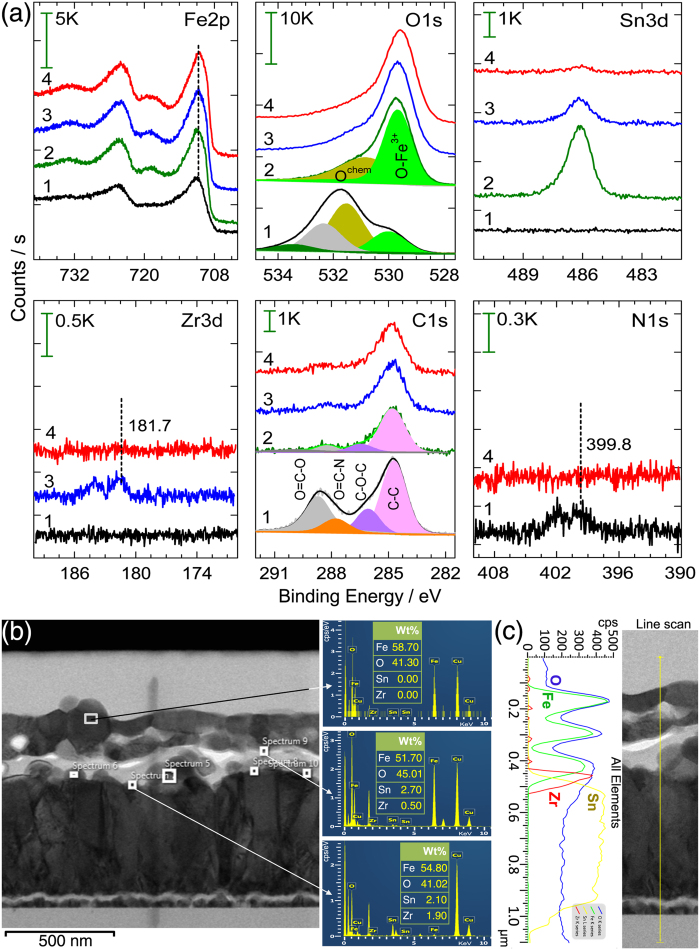
(**a**) High resolution XPS spectra of Fe2p, O1s, Sn3d, and Zr3d for as-grown (*Curve* 1: FZC) and annealed (*Curve* 2: F, 3: FZ, 4: FZC) films, (**b**) Cross-sectional TEM micrograph of annealed FZC sample with point EDS spectra revealing the % compositions of Fe, O, Sn and Zr elements, and (**c**) TEM-EDS line profile revealing the gradual diffusion doping of Sn and Zr ions.

The chemical compositions in the Fe_2_O_3_ films are studied by XPS. Figure S5 shows the survey XPS spectra of surfactant-mediated Fe and Fe_2_O_3_ films synthesized on FTO using ZrO_2_ UL, revealing individual components. Table S2 shows the corresponding elemental quantifications. Figure 4a shows the high resolution XPS spectra of Fe2p, O1s, Sn3d, Zr3d, C1s, and N1s components. Broad Fe2p_3/2_ peak and nature of satellite peak confirms the +2 oxidation state for Fe in as-grown film, which can exist in FeO or Fe_3_O_4_ upon air-exposure. Fe 2p_3/2_ BE peak at 710.8 ± 0.1 eV in annealed film confirms the +3 oxidation state of Fe and phase-pure formation of α-Fe_2_O_3_^34^. The O1s peak at ~530 eV originates from the Fe_2_O_3_ moiety and the one at ~531 eV is due to adsorbed hydroxyl groups^35^. The XPS signal of Sn for sample F originate from the diffusion of Sn^4+^ ions from the FTO substrate during HT-annealing^36^. It is generally accepted that PEC response is very sensitive to the Sn doping^37^. The XPS signals of Zr originate from the diffusion of Zr^4+^ ions from ZrO_2_ UL^10^. It is clear from XPS spectra that the Sn signal is higher for the sample F showing highest surface Sn content (1.4 at.%), which is dropped to 0.5 at.% and 0.1 at.% for FZ and FZC samples, respectively. However, it is likely that the Sn is incorporated into the bulk part of hematite considering the higher film thicknesses of FZ and FZC samples. Moreover, it is also possible that ZrO_2_ UL on the FTO may have partially blocked the release of Sn^4+^ ions from FTO during HT-annealing. Although 0.4 at.% Zr is detected for FZ sample, it is negligibly small for FZC upon use of CTAB. These observations suggest that the diffused Zr ions could have explicably incorporated into the bulk of hematite and did not reach the surface due to higher film thickness. The as-grown FZC sample showed ~41 at.% carbon (mainly due to surfactant) compared to that for annealed FZC sample (19–20 at.%, which is atmospheric in origin). The C1s signal of as-grown sample reveals four peaks at ~284.8, 286.1, 287.8, and 288.7 eV, which are attributed to C−C/C−H (from atmospheric carbon) C−O–C, O=C−N, and O−C=O groups^38^. Besides the carbon peak, the presence of N1s (1.6 at. %) signal clearly confirms the incorporation of CTAB surfactant in the as-grown film. To gain insight into the diffusion doping of Zr^4+^ and Sn^4+^ ions into the bulk of hematite, the point EDS spectra are obtained for the porous FZC sample that reveals the elemental composition at selective regions of the cross-section. As shown in Fig. 4b, the concentration of diffused Zr ions is very small in comparison to the Sn ions. The FZC sample featured 2.7 wt.% Sn in the hematite bulk with no Sn near the surface. The Zr contents of 0.5 wt.% and 1.9 wt.% are observed in the bulk of hematite and at the FTO interface, respectively. This also confirms the presence of Zr in the bulk of FZC sample. As it is difficult to observe the ZrO_2_ UL (upon HT-annealing) from cross-sectional SEM image, a line profile analysis at the cross-section of FIB-cut FZC sample is performed. Figure 4c shows a TEM-EDS line profile of FIB-cut FZC sample. Line profile reveals the gradual incorporation/diffusion of Sn^4+^ ions from FTO and Zr^4+^ ions from ZrO_2_ UL into the hematite lattice with a larger portion of Zr ions at the interface of hematite and FTO, suggesting that a thin underlayer of ZrO_2_ still remains. However, the thickness of remaining UL may vary over the entire interface.

**Figure 5:**
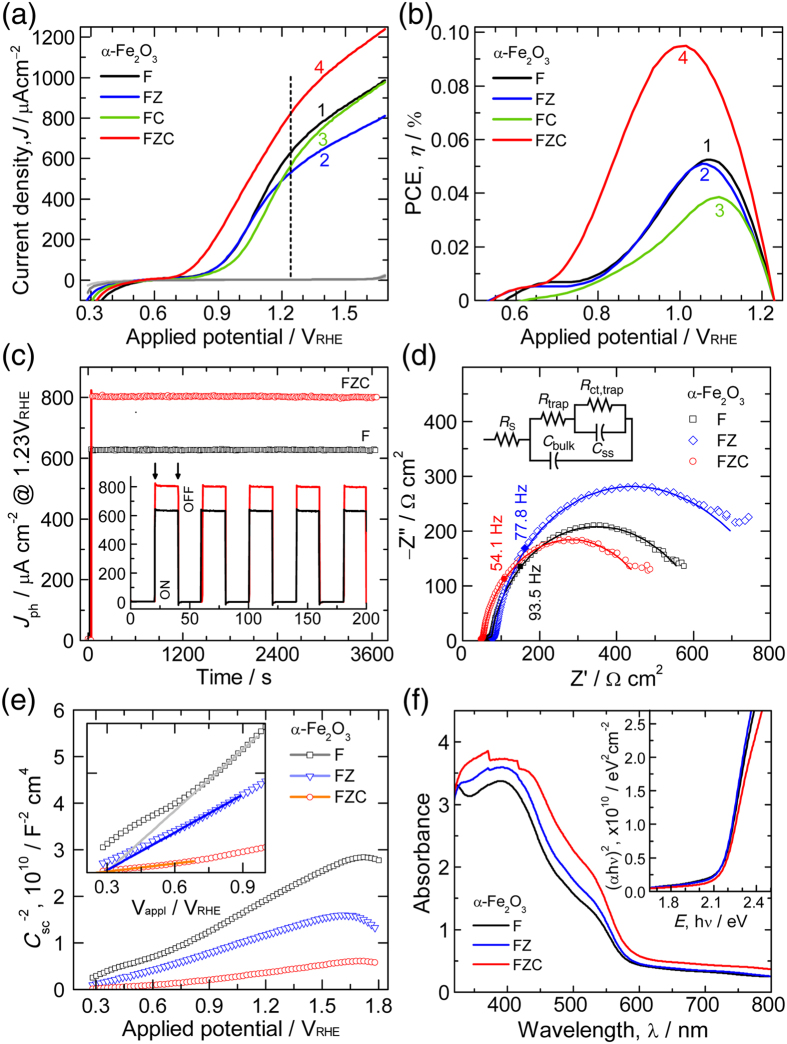
(**a**) *J*−*V* and (**b**) PCE curves (1: F, 2: FZ, 3: FC, and 4: FZC), (**c**) Long-term stability test @ 1.23 V vs. RHE and the chronoamperometry responses under ON-OFF light switching (inset), (**d**) Nyquist and (**e**) MS plots of Fe_2_O_3_ photoanodes. EIS and MS data are obtained at 1.23 V_RHE_ under illumination and under dark condition, respectively. Inset shows the equivalent electrochemical circuit used to fit the Nyquist plots. Scan rate: 20 mV s^–1^; Illumination: 1 sun; Electrolyte: 1 M NaOH. (**f**) UV-Vis absorption spectra and the corresponding band gap energy plots (inset) of Fe_2_O_3_ photoanodes.

The PEC performances of planar and porous Fe_2_O_3_ films are evaluated as photoanodes for solar water splitting. Initially, the concentration of CTAB in the sulfate electrolyte is optimized by varying the CTAB concentrations from 2 to 20 mM and checking the photocurrent performance of hematite photoanodes fabricated using those concentrations as shown in Fig. S6. In all the cases, no photocurrent improvement is observed with respect to the base hematite photoanode fabricated without CTAB. The film homogeneity is higher for CTAB concentrations greater than 10 mM. As seen from figure, the FC photoanode fabricated using 10 mM CTAB showed comparable photocurrent response when compared with bare F photoanode. Therefore, a 10 mM CTAB concentration is selected for further study. Additionally, we noticed that a remarkable enhancement in the photocurrent is observed only upon the use of 10 mM CTAB along with ZrO_2_ underlayer on FTO. Figure 5a shows the photocurrent-potential (*J*–*V*) curves of F, FZ, FC, and FZC photoanodes. The photocurrent density (*J*_ph_) of sample F at 1.23 V_RHE_ is decreased from 625 to 525 μA cm^−2^ upon use of ZrO_2_ UL showing no discernible change in onset potential (*V*_onset_). This decrease could be due to higher incorporation of Zr ions into the hematite. Similar effect is observed for Zr-doped Fe_2_O_3_ nanorod-type photoanode fabricated by hydrothermal method^15^. Higher concentration of dopant would provide more defect-scattering/recombination properties inhibiting the increased separation efficiency thereby affecting the photocurrent response of hematite^15^,^39^. The *V*_onset_ values of the respective photoanodes are *ca.* 0.889 and 0.845 V_RHE_. The *J*_ph_ of FC sample showed lower photocurrent that originates from the inhomogeneity of the hematite film. The porous FZC photoanode synthesized using a ZrO_2_ UL and CTAB surfactant depicted remarkable improvement in both *J*_ph_ and *V*_onset_ values. The photoanode showed optimum *J*_ph_ (812 μA cm^−2^) at 1.23 V_RHE_ and significantly lower *V*_onset_ value (*ca*. 0.76 V_RHE_), which is higher by a magnitude of 129 mV compared to the planar F sample. Here, the CTAB helps in creating porous channels for easier diffusion of Sn^4+^ or Zr^4+^ ions into the hematite lattice while Zr ions helps improve the interface properties of hematite. Thus, ZrO_2_ UL and CTAB-mediated porosity synergistically boosted the hematite performance. Higher *J*_ph_ value for FZC film than the recently reported mesoporous film using silica templates (610 μA cm^−2^)^40^, indicates the superior activity of our porous films. Contrary to the FZ sample, asgrown FZC sample is relatively thicker and gradually turns into porous during HT-annealing, as a result of which the Zr ions get incorporated homogeneously into hematite. Figure 5b shows the photocurrent conversion efficiency (PCE)^41^, *η* that reveals a trade-off between the power output required for solar water splitting and the external power supplied to convert the light energy. This trade-off provides a maximum *η* value of 0.095% for porous FZC sample at 1.01 V_RHE_, which is 103% higher than that of F sample. Such superior performance is collectively attributed to the nanoporous architecture and improved interface properties (discussed later). To further analyze the photoelectrochemical stability, the chronoamperometry curves of F and FZC photoanodes are recorded at 1.23 V_RHE_ for 3600 s under one sun illumination as shown in Fig. 5c. The photocurrents of both the photoanodes are stable for a significantly longer time. FZC photoanode, however, showed a slight decrease in average *J*_ph_ value after an hour of illumination (~0.7% decrease). Inset of Fig. 5c shows the current responses of F and FZC photoanodes under ON-OFF switching of illumination.

The synergistic improvement in PEC activity induced by Sn or Zr-doping and surfactant-mediated nanoporous architecture is further verified by investigating the charge-transfer processes using electrochemical impedance spectroscopy (EIS) techniques. Figure 5d shows the Nyquist plots for all Fe_2_O_3_ photoanodes. The Nyquist curves are fitted using circuit elements consisting of one resistor and two RC circuits in parallel^42^. The series resistance (*R*_s_), generally meant for FTO resistance (since the resistances of the external contacts are negligibly small), is very high for sample F revealing a loss of FTO conductivity (Table 1). No significant change in *R*_s_ for FZ sample is observed although it is decreased slightly compared to F sample. Considerable decrease in *R*_s_ value for FZC sample means that both ZrO_2_ UL and the carbonaceous entities from the CTAB surfactant partially block the release of Sn ions from FTO at the early stage of HT-annealing process, thereby reducing the rapid loss in FTO conductivity. Thus, ZrO_2_ UL and CTAB together improved the interface properties of Fe_2_O_3_ keeping minimal loss in FTO conductivity. Low charge transfer resistance (*R*_ct_) for FZC (Table 1) can primarily be interpreted by a significantly enhanced conductivity in Fe_2_O_3_ due to doping: largely due to Sn^4+^ ions (from FTO) and partially due to Zr^4+^ ions (from ZrO_2_ UL). It also meant that nanoporous architecture of the film helped facilitate the charge transfer of holes to the electrolyte species efficiently leading to the photocurrent improvement^43^. The surface state capacitance (*C*_ss_) governs the presence of surface or defect states (whose chemical identity is Fe-O*) that are formed due to oxygen vacancies^17^. The surface states with or without coupling with protons have been reported to play pivotal role by determining the thermodynamics of surface hole-trapping in a first step, followed by controlling the hematite water oxidation activity. In other words, *C*_ss_ has been related to the charging of surface states, which builds up due to formation of Fe-OH_x_ intermediate state. Several groups have shown that peak value of *C*_ss_ coincides with the photocurrent *V*_onset_^42^,^44^. Relatively high *C*_ss_ value for FZC sample indicates that the multiplied influxes of holes (available for charge transfer to the electrolyte) are driven by the large nanoporous architecture of Fe_2_O_3_ photoanode. Higher *C*_ss_ value is consistent with the cathodic shift in *V*_onset_ for Fe_2_O_3_ films^43^. In our previous study, it is shown that higher crystalline orientation along (104) plane is consistent with the higher degree of surface states, which resulted in higher *C*_ss_ value^37^. Similar trend is also observed in the present study. As seen from XRD study, the (104) plane of hematite associated with surface states shows pronounced intensity for FZC sample. Additionally, the activation energy of electron transfer (hopping) in such photoanode is expected to dramatically decrease because the electron carriers are favorably repelled (do not get trapped) in the presence of low-valent Zr^4+ ^17, which significantly improves the electron mobility. Thus, large number of accumulated holes facilitates the water oxidation process leading to early photocurrent onset (as seen from PEC study). Miao *et al.* observed higher *C*_ss_ value, which is consistent with the cathodic shift in *V*_onset_ of Fe_2_O_3_ films^43^. Recently, Li *et al.* showed prominent PEC performance for hematite nanorods consisting a thin amorphous TiO_2_ layer (as a defect-creating layer) and the improvement is attributed to the accelerated hole-transfer via surface trap at the hematite-electrolyte interface^45^. Wang *et al.* reported that deliberate creation of lattice defects in hematite enhanced the PEC performance causing cathodic shift in the photocurrent onset^46^. The findings from these studies affirm the cathodic shift observed for our film. Additionally, electron lifetime (*τ*_e_) can be correlated with the characteristic maximum frequency peak (*f*_max_) in the Bode phase plot by a relation:*τ*_e_ = 1/(2π*f*_max_)^47^. The decrement in *f*_max_ value from 93.5 to 54.1 Hz suggests that porous FZC sample possesses a 2-fold higher electron lifetime (*τ*_e_ = 2.94 ms) than sample F (Fig. S7). The similar electron life time values estimated from Bode phase plot are reported for other hematite photoanodes^48^,^49^. The superior performance from FZC sample originates from the efficient hole transfer to the interface of photoanode-electrolyte junction^50^. Therefore, a low recombination rate is highly desired to achieve high charge collection efficiency, eventually leading to high solar conversion efficiency. Above results are in line with the several studies, which have hypothesized that the surface states are important for efficient water oxidation reaction^50^,^51^,^52^,^53^,^54^. The charge carrier concentration or donor density (*N*_D_) is estimated from Mott-Schottky plot (Fig. 5e). Lower *N*_D_ value for sample F meant that conductivity contribution comers from the diffused Sn ions alone in a relatively less thick film. Table 1 reveals almost two- and seven-fold increments in the *N*_D_ values for the FZ and FZC samples, respectively. Higher *N*_D_ value for FZC is due to enrichment of electrons from Zr and Sn dopants in relatively thicker film. Large portion of these ions remain into the bulk of hematite. When added to hematite upon HT-annealing via diffusion, Zr ion donate an extra electron to reside on the Fe cation in the hematite lattice and does not affect the Fe to Fe mode of electron transport, suggesting that ZrO_2_ UL enriches the hematite conductivity^17^. Consistent *N*_D_ values on the order of 10^19^–10^20^ cm^−3^ are reported for hematite^55^. Higher *N*_D_ reduces the width of space charge layer, which helps in charge separation and improves the photocurrent in FZC photoanode. In general, the increase in charge carrier density (*N*_D_ or *n*) is associated with higher electrical conductivity (*σ*) by a relation: *σ* = *enμ*, where *e* is the electronic charge and *μ* is the charge carrier mobility. Increased mobility is highly favorable for improving charge transport and charge separation processes in water splitting. Moreover, higher *N*_D_ in porous hematite raises the Fermi level of Fe_2_O_3_ toward its conduction band, which leads to more significant band bending in the space charge region than that of flat hematite due to a larger difference between the Fermi level of Fe_2_O_3_ and the redox potential of the electrolyte. The enhanced electric field in the space charge layer facilitates the charge separation and slows the charge recombination rate. The positive shift in *V*_fb_ to 0.498 V_RHE_ for nanoporous FZC photoanode indicates the decreased band bending, which has a major effect on improving the photocurrent. This effect can be ascribed to the improved photoanode-electrolyte interface, which facilitates the efficient charges transfer^56^. Thus, significant enhancement in *J*_ph_ is credited to its increased charge transport because of higher conductivity and charge separation.

Figure 5f shows the UV-vis absorbance spectra of F, FZ and FZC photoanodes. The absorbance of FZC sample is increased noticeably in the wavelength range between 350 and 600 nm in comparison to F and FZ samples. The increased absorbance of FZC sample can partly be attributed to the higher film thickness in comparison to F and FZ samples. Increase in absorption for increasing film thickness directly follows from the Beer-Lambert’s law. Moreover, it is also true that the light penetration depth is inversely proportional to the optical absorption. Therefore, although the optical absorption is higher, the absorbed light (photo-generated electron-hole pairs) may not be fully utilized to contribute to the photocurrent enhancement because of a short hole diffusion length in hematite. As a result the photo-generated charge carriers are recombined, resulting in a poor photocurrent^24^. The film thickness of planar F sample deposited at 90s (in our previous work^24^) is comparable to that of FZC sample with slightly higher absorbance; however, the resulting photocurrent is four-times lower. Therefore, it’s the porosity in FZC sample that causes multiple light scatterings inside, which helps in effective utilization of absorbed light toward photocurrent enhancement. However, the contribution of higher photocurrent for FZC sample comes from its increased conductivity. The band edge for the FZ sample is shifted slightly towards a higher wavelength, suggesting a slight shift in the band edge. The shift is quite noticeable for the FZC sample. However, no significant changes in the estimated band gap energy (*E*_g_) values are observed (*E*_g_ = 2.15–2.17 eV). The negligible effect of Zr doping on the band gap has been reported for α-Fe_2_O_3_ films co-doped with other cations such as Ti^4+^, Sn^4+ ^15. The Sn doping has been reported to affect the absorption in hematite^57^,^58^.

**Figure 6:**
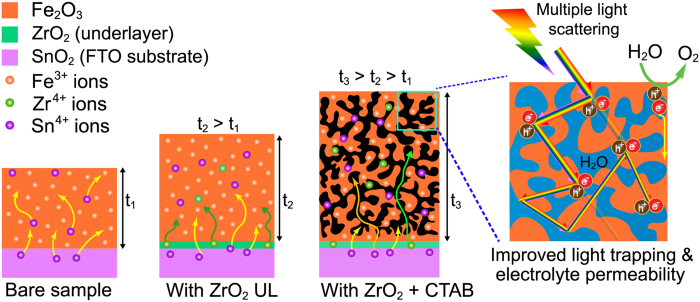
Diffusion doping mechanism of Sn and Zr ions in hematite for different synthesis conditions.

Figure 6 shows the diffusion doping mechanism of Sn^4+^ and Zr^4+^ ions during hematite formation at HT-annealing. For relatively thin and compact bare sample, HT-annealing causes partial release of Sn^4+^ ions from FTO, which readily diffuse into the Fe_2_O_3_ by migrating toward the crystallizing Fe_2_O_3_ lattice. In the case of Fe_2_O_3_ fabricated using a ZrO_2_ UL, Sn^4+^ and Zr^4+^ ions concurrently incorporate into hematite as a result of solid state reaction between the crystallizing lattice Fe_2_O_3_ (slightly thicker film) and ZrO_2_ UL as well as FTO, transferring one electron each to the Fe sites. The Zr ion maintains a charge-neutrality without forming additional energy levels due to its low ionization energy as well as its instability. The electronic conduction occurs by hopping of electrons via Fe-to-Fe sites. Considering the compact thicker films, the diffusion rate of Sn^4+^ ions via grain boundaries is limited. The Zr content in hematite is relatively small because the concentration as well as diffusion rate of Zr^4+^ ions from ZrO_2_ UL is lower compared to the Sn^4+^ ions from FTO substrate. In the case of FZ sample, the thickness (so does the absorbance) is slightly higher than that of sample F. Although the conductivity of FZ sample is higher (as seen from the Mott-Schottky plot), the absorbed light (photo-generated electron-hole pairs) is not fully utilized for the photocurrent enhancement since hematite has a short hole diffusion length. Large portion of photogenerated electron-hole pairs are annihilated by recombination. In the third case (more thicker FZC films), the surfactant molecules are annihilated during HT-annealing, creating number of non-resistant porous pathways for gradual diffusion of Sn^4+^ and Zr^4+^ ions into the crystallizing Fe_2_O_3_ lattice. The XPS-detected presence of Sn^4+^/Zr^4+^ ions on the surface of FZC sample is lower because most of the diffused ions are in the bulk of hematite and do not reach the surface due to higher film thickness. However, due to porous nanoarchitecture of FZC sample, the photo-generated electron-hole pairs are multiplied despite of higher film thickness, thus enhancing the utilization of absorbed photons to great extent. Additionally, porous architecture also helps in easier incorporation of dopants into the hematite lattice, thereby increasing its conductivity. As a result, the donor concentration in hematite is dramatically improved. The formation of nanoporous architecture is best suited for scattering functions inside the photoanode and could lengthen the light pathways, thereby improving light harvesting in the hematite. The nanoporous architecture enables a maximized absorption in regions where the photo-generated minority charge carriers reach the surface and minimize the wasted absorption in regions where they could be lost due to recombination. Thus, the utilization of surfactant helped improve light trapping as well as electrolyte permeability. Such hematite with large porous area and improved charge collection properties would be ideal for water splitting reactions. The photocurrent of such porous Fe_2_O_3_ photoanodes could further be improved via *ex-situ* Sn^4+^-doping considering the fact that the doped Sn content in hematite is not optimum. Additionally, use of co-catalysts would further improve the performance.

In summary, we have demonstrated the viability of nanoporous architecture-containing hematite photoanodes for efficient solar water splitting. The photoanodes were prepared via a facile PRED method by incorporating a cationic CTAB surfactant in the electrolyte and use of ZrO_2_ underlayer on FTO. High temperature-annealing at 800 °C was employed to induce Sn^4+^ and Zr^4+^ doping into the hematite. In a dramatic change, the ZrO_2_ underlayer and CTAB collectively transformed the crystalline orientation of hematite by promoting growth along (104) while suppressing the growth along (110) direction. The nanoporous hematite photoanode exhibited substantially higher photocurrent with cathodic shift in onset potential compared to a planar photoanode. Such impressive performance was attributed to the enriched carrier concentration, improved light trapping, efficient charge transfer, hematite crystalline orientation and the surface states. We believe that these findings would open up new opportunities for design and fabrication of high performance hematite photoanodes for PEC water splitting.

## Methods

### Chemicals and materials

Chemical reagents such as iron (II) sulfate heptahydrate (FeSO_4_·7H_2_O, ≥98%, Alfa Aesar), ascorbic acid, amidosulfonic acid, boric acid, zirconyl nitrate solution (ZrO(NO_3_)_2_, 35 wt.% in dilute nitric acid, ≥99%, Aldrich), titanium diisopropoxide bis(acetylacetonate) (C_16_H_28_O_6_Ti, 75 wt.% in isopropanol, Aldrich), cetyltrimethylammonium bromide (CTAB, C_19_H_42_BrN, ≥98.0%, Aldrich), absolute ethanol, acetone, and ethanol were used as-is without further purification. All the solutions were prepared from high-purity deionized water (Scholar type, Human Power I + Corp., resistivity <18 MΩ cm). Iron films (to be converted to iron oxide upon annealing) were prepared via PRED on conducting fluorine doped tin oxide (FTO)-coated glass substrates (TEC45, 1 cm × 2.5 cm, 10−15  Ω cm^−1^).

### Materials synthesis

Iron films (to be converted to iron oxide) were prepared via pulse reverse electrodeposition (PRED) method on clean FTO substrates. The cleaning of FTO substrates, preparation of electrolyte and the process parameters of PRED (square wave pulse amplitude of 10 V [−6/ + 4 V], duty cycle of 20%, pulse period of 10 ms and deposition time of 45 s) were accomplished according to our previous study^24^. A sulfate bath with or without CTAB was served as the electrolyte for PRED. The concentration of CTAB in the sulfate bath was optimized to be 10 mM based on the photocurrent performance and homogeneity of Fe_2_O_3_ photoanodes fabricated using different CTAB concentrations viz. 2, 5, 10, 15 and 20 mM. The appropriate amount of surfactant was dissolved in a 50 mL sulfate bath while stirring for 10 min followed by ultrasonic agitation for 30 min to ensure a clear homogeneous solution. Solutions were made afresh for all the PRED experiments. Along with surfactant, the effect of ZrO_2_ UL on FTO was also studied. The ZrO_2_ UL was obtained on FTO by spin-coating (2500 rpm for 30 s) about 150 μL (75 μL, two times) of 10 mM zirconium precursor solution prepared in absolute ethanol. The spin-coated ZrO_2_/FTO substrates were then quenched in air after being soaked at 250 °C for 30 min. The amorphous ZrO_2_ layer containing FTO substrates were used to grow iron films via PRED with and without CTAB. All the PRED experiments were performed under identical conditions at room temperature and at least three electrodes of each condition were prepared for checking reproducibility of the films and their performance. As-grown films were blackish in color and reflected light. After deposition, the resultant films were rinsed with a copious amount of deionized water and dried under a nitrogen stream. As-grown films were then converted to iron oxide by a one-step HT-annealing method that involves quenching of films in air after soaking them in a box furnace at 800 °C for 13.5 min^59^. The iron oxide films appeared dark reddish brown, depending on the film thickness.

### Characterization of materials

The representative Fe_2_O_3_ samples were comprehensively characterized using several techniques. Structural analysis was performed using synchrotron XRD at both 9C and 5A beamlines of the Pohang Light Source II (PLS-II) in Korea. Conventional theta-two theta (θ–2θ) scans were carried out with X-rays of 8.9 keV (λ = 0.1393 nm) at the 9C beamline. For line profile analysis of the Williamson-Hall plots, 2-dimensional (2D) XRD patterns from the photoanodes were recorded using a 2D detector (MAR345 image plate) at the 5A beamline and a probing X-ray energy of 16.2 keV (λ = 0.0765 nm). The incident angle and sample to detector distance (SDD) was 3 degrees and 366 mm, respectively. Subsequently, the 2D XRD pattern was converted into a line profile via the FIT2D program^60^. X-ray absorption fine structure (XAFS) measurements were conducted on the 7D beamline of the Pohang Accelerator Laboratory (PLS-II, 3.0 GeV). The synchrotron radiation was monochromatized using Si(111) double crystal monochromators. Spectra for the Fe K-edge (*E*_0_ = 7112 eV) were taken under fluorescence mode at room temperature. X-ray absorption near edge (XANES) spectra and extended X-ray absorption structure (EXAFS) functions were obtained only for the representative samples. The incident beam was detuned by 15% at 7112 eV to attenuate the flux from higher order Bragg diffractions of silicon crystals in the monochromator. Its intensity was monitored using a He-filled IC SPEC ionization chamber and the fluorescence signal from the sample was measured with a PIPS (passivated implanted planar silicon) detector. ATHENA in the IFEFFIT suite of programs was used to analyze the obtained data for the local Fe structure in the Fe_2_O_3_ photoanodes synthesized using ZrO_2_ UL with and without surfactant^61^. The thickness of the ZrO_2_ UL was measured using a variable angle spectroscopic ellipsometer (M-2000V, J.A. Woollam Co., Inc.). To estimate the thickness of ZrO_2_ UL, spectroscopic ellipsometry analysis of ZrO_2_ film coated on soda lime glass was performed using a cleaned soda lime glass as a reference. The samples were analyzed in the spectral range of 245–1690 nm at variable angles of incidence (ϕ = 45–70°). The thickness of the ZrO_2_ UL was estimated using s wave [I_s_ = sin(2Ψ)*sin(Δ)] and p wave [I_p_ = sin(2Ψ)*cos(Δ)] components with the help of a computer-aided program. The surface and cross-sectional morphologies of all the Fe_2_O_3_ films were examined on a Field Emission Scanning Electron Microscope (FESEM) (SUPRA 40VP, Carl Zeiss, Germany), equipped with an X-ray energy dispersive spectrometer (EDS). The chemical state and elemental quantification of the freshly synthesized iron oxide samples were performed using X-ray photoelectron spectroscopy (XPS) on a PHI Quantera II spectrometer equipped with a monochromatic Al K*α* X-ray source (*hν* = 1486.6 eV, 50 W, and 15 kV). The detection angle relative to the substrate surface was 45°. Wide survey spectra (binding energy, BE: 1200−0 eV) were recorded using an X-ray spot size of 200 μm at room temperature with an analyzer pass energy of 280 eV and an energy step size of 1 eV. High resolution spectra in the region of interest were acquired with pass energy of 55 eV and step size of 0.1 eV. XPS data processing including peak deconvolution was performed with the help of an XPS Peak-fit program using Shirley background subtraction and an iterative least-squares optimization algorithm. The high resolution XPS spectra were calibrated by referencing the maximum of adventitious hydrocarbon peak at 284.8 eV. Deconvolution of the C1s and O1s peaks was performed according to their chemical environments. To observe Sn doping across the representative Fe_2_O_3_/FTO photoanodes, full cross-sectional TEM samples were prepared with a dual-beam focused ion beam (FIB, Helios NanoLab, FEI) using a Ga+ ion beam source operating at 30 kV. Elemental analysis and elemental line profile mapping across the cross-sections were performed with a field-emission transmission electron microscope (JEM-2100F HR, JEOL) operating at 200 kV, equipped with an energy dispersive spectrometer (EDS). UV-vis absorption study in the wavelength range from 350 to 800 nm was performed using a dual beam spectrophotometer (Shimadzu, UV-2600 series).

### Photoelectrochemical measurement

The water oxidation performance of the Fe_2_O_3_ photoanodes was measured in a PEC cell comprising a three-arm glass compartment with a circular quartz window for light illumination. The PEC cell components included a Fe_2_O_3_/FTO photoanode as the working electrode, Pt wire as the counter electrode, Ag/AgCl (saturated with KCl) as the reference electrode, and 1 M NaOH as the electrolyte. A standard illumination of simulated 1 sun (100 mW cm^−2^, AM1.5G) was provided using a solar simulator (Abet Technologies). All potentials mentioned in the work were originally measured with reference to the Ag/AgCl electrode (sat. KCl), and were revised to the reversible hydrogen electrode (RHE) scale using Nernst equation (2)^12^:


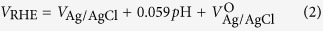


where *V*_RHE_ was the converted potential vs. RHE, 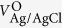 = 0.1976 V at 25 °C, and *V*_Ag/AgCl_ was the experimental potential against the Ag/AgCl electrode. The current–voltage (*J*−*V*), electrochemical impedance spectroscopy (EIS), and Mott–Schottky (MS) studies were performed using a potentiostat (Ivium, Netherland) equipped with an electrochemical interface and impedance analyzer facility. EIS study was performed, to investigate the charge-transport properties, under 1 sun illumination at 1.23 vs. RHE (V_RHE_). The experimental EIS (real *vs*. imaginary impedance) data was validated using the Kramers–Kronig transform test and fitted to a suitable equivalent circuit model using the ZView (Scribner Associates Inc.) program. MS (*C*_sc_^−2^ *vs. V*) measurements were performed under dark conditions with an applied DC potential window of −0.6 to 0.7 V vs. Ag/AgCl at 0.5 kHz AC frequency. The amplitude of the AC potential was 10 mV in both EIS and MS measurements. The carrier concentration or donor density (*N*_D_) was estimated from the slope in the quasi- linear region of MS plot near the flat band potential (*V*_fb_) according to the MS analysis^62^.

## Additional Information

**How to cite this article**: Shinde, P. S. *et al.* A Synergistic Effect of Surfactant and ZrO_2_ Underlayer on Photocurrent Enhancement and Cathodic Shift of Nanoporous Fe_2_O_3_ Photoanode. *Sci. Rep.* **6**, 32436; doi: 10.1038/srep32436 (2016).

## Supplementary Material

Supplementary Information

## Figures and Tables

**Table 1:** EIS and MS parameters obtained for Fe_2_O_3_ photoanodes fabricated with or without the use of ZrO_2_ UL and surfactant.

Samples/Parameters	*R*_s_ Ω cm^2^	*R*_ct,trap_ Ω cm^2^	*C*_ss [CPE]_ μF	*τ*_e_ ms	*V*_onset_ V_RHE_	*V*_fb_ V_RHE_	*N*_D_, × 10^19^ cm^−3^
F	60.12	596.3	7.80	1.70	0.889	0.294	8.64
FZ	56.60	806.4	10.86	2.05	0.845	0.280	13.95
FZC	47.34	449.0	41.00	2.94	0.760	0.262	62.65
