# Supplementary material for: A Synergistic Effect of Surfactant and ZrO2 Underlayer on Photocurrent Enhancement and Cathodic Shift of Nanoporous Fe2O3 Photoanode
Source: Sci Rep. 2016 Aug 31;6:32436. doi: 10.1038/srep32436 (PMC5006030; doi:10.1038/srep32436)
Supplement: Supplementary Information [file srep32436-s1.pdf]

Supplementary Information

A Synergistic Effect of Surfactant and ZrO_2 Underlayer on Photocurrent Enhancement and Cathodic Shift of Nanoporous Fe_2O_3 Photoanode

Pravin S. Shinde,[1] *Su Yong Lee,*[2] *Sun Hee Choi,*[2] *Hyun Hwi Lee,*[2] *Jungho Ryu,*[3] *and Jum Suk Jang**[1]

[1] Division of Biotechnology, Division of Biotechnology, Safety, Environment and Life Science Institute, College of Environmental and Bioresource Sciences, Chonbuk National University, Iksan 570-752, Republic of Korea.

[2] Pohang Accelerator Laboratory, Pohang University of Science and Technology (POSTECH), Pohang 790-784, Republic of Korea.

[3] Mineral Resources Research Division, Korea Institute of Geoscience and Mineral Resources (KIGMR), Daejeon 305-350, Republic of Korea.

* Corresponding author.

E-mail address: jangjs75@jbnu.ac.kr (JSJ)

Table S1. Crystallite size and micro strain of the Fe_2O_3 photoanodes according to the synthesis conditions.

Photoanode	D (nm)	ε_{μ} ($\times 10^{-4}$)
F	59.6	4.6
FZ	61.9	4.3
FC	61.9	10.8
FZC	63.9	6.2

Table S2. Elemental quantification of surfactant-mediated as-grown and annealed Fe_2O_3 films.

Samples/	**Fe 2p**		**O 1s**		**Sn 3d5**		**Zr 3d**		**N1s**		**Br3d**		**C1s**	
Parameters	BE	at.%	BE	at.%	BE	at.%	BE	at.%	BE	at.%	BE	at.%	BE	at.%
F	710.8	32.7	529.8	66.0	486.3	1.4	--	--	--	--	--	--	--	--
FZ	710.8	32.9	529.7	66.3	486.2	0.5	183.6	0.4	--	--	--	--	--	--
FZC	710.8	33.8	529.7	66.1	486.2	0.1	183.6	<0.1	--	--	--	--	--	--
FZC (As-grown)	710.8	11.2	529.9	46.1	--	--	--	--	486.3	1.6	284.8	<0.1	284.8	41.1

Note: The peak values were calibrated with reference to adventitious carbon at 284.8 eV. About 19-20 at% carbon (C1s) is detected in annealed samples due to atmospheric impurities and it is not considered in the above table.

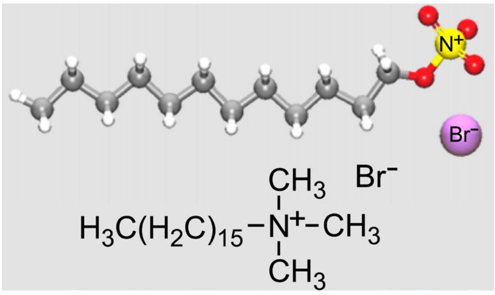

Figure S1. Structure of CTAB surfactant molecule

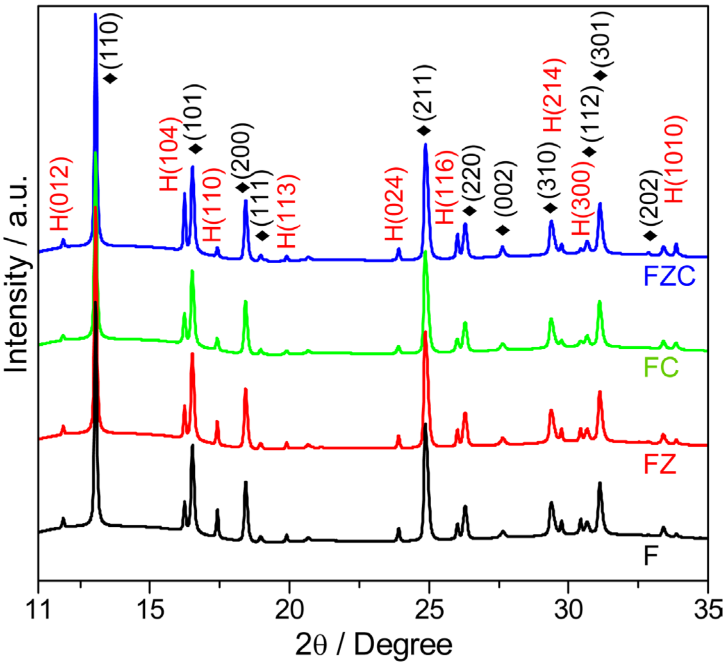

Figure S2. The line profiles converted from the 2D XRD patterns (λ=0.0765 nm) of Fe_2O_3 photoanodes fabricated using UL and surfactant. The letters 'H' and '♦' along with the (*hkl*) planes are used to designate the corresponding reflections of hematite and FTO substrate, respectively.

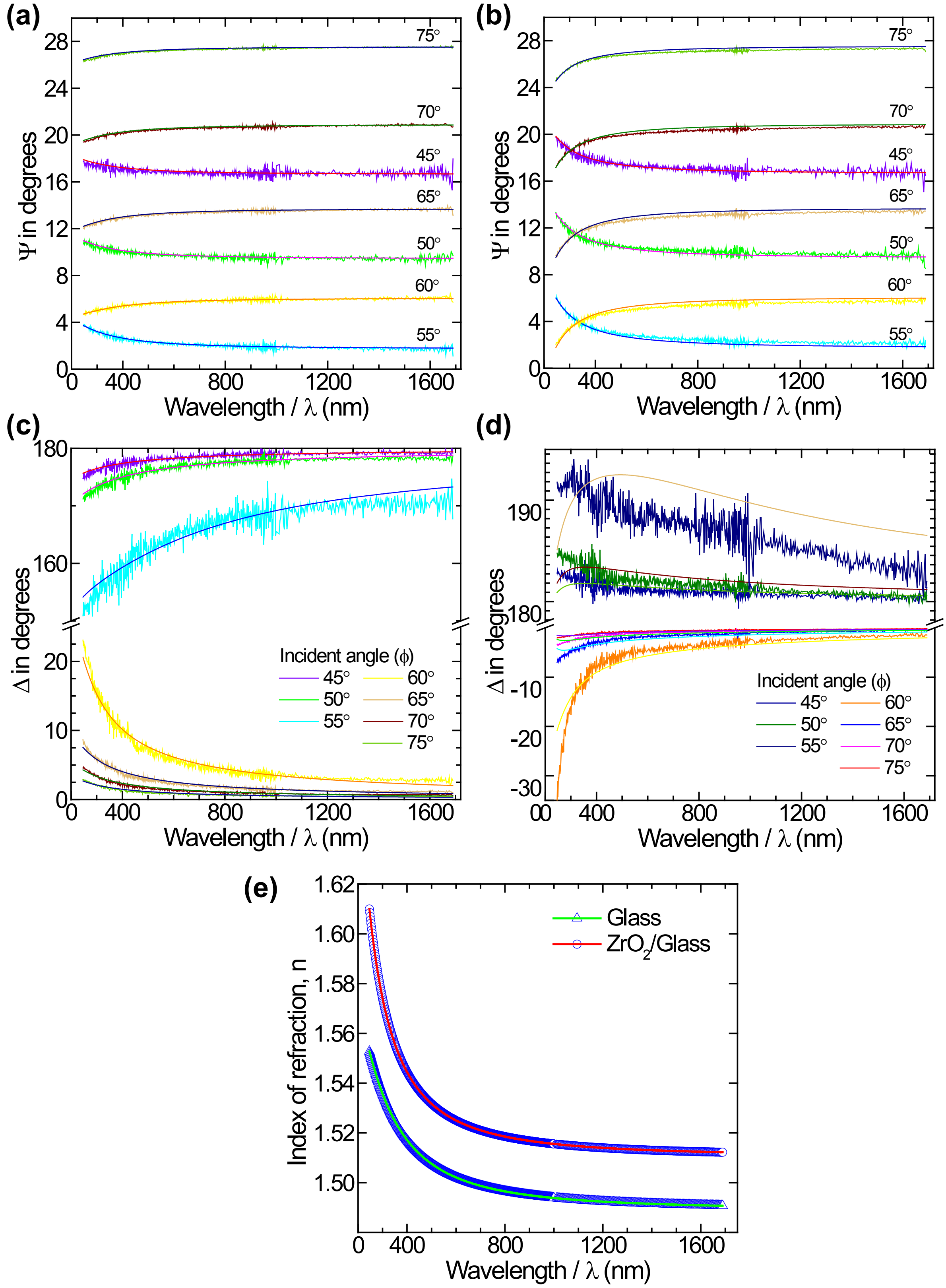

Figure S3. Plots of Ψ and Δ with their Cauchy fits for (a,c) glass and (b,d) ZrO_2 underlayer coated-glass samples for variable angles of incidence (ϕ= 45–75°); (e) Refractive index plot.

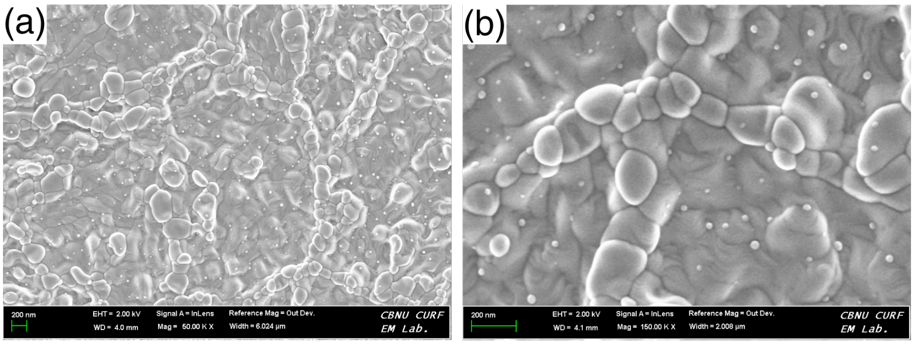

Figure S4. FESEM surface images of Fe_2O_3 photoanode fabricated using only surfactant at (a) 50 KX and (b) 150 KX magnifications.

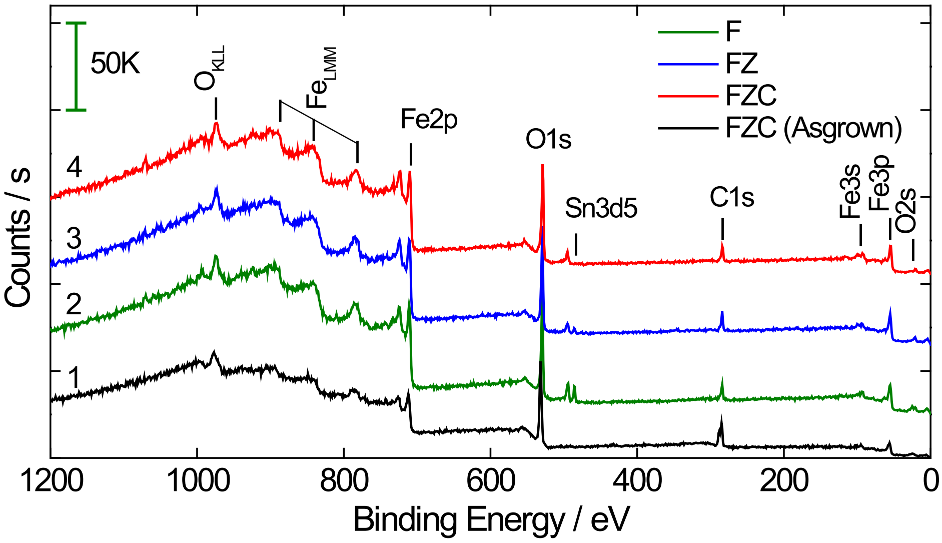

Figure S5. Survey XPS spectra of different films synthesized on FTO using ZrO_2 UL and surfactant, *Curves*- 1: FZC (As-grown), 2: F, 3: FZ, 4: FZC.

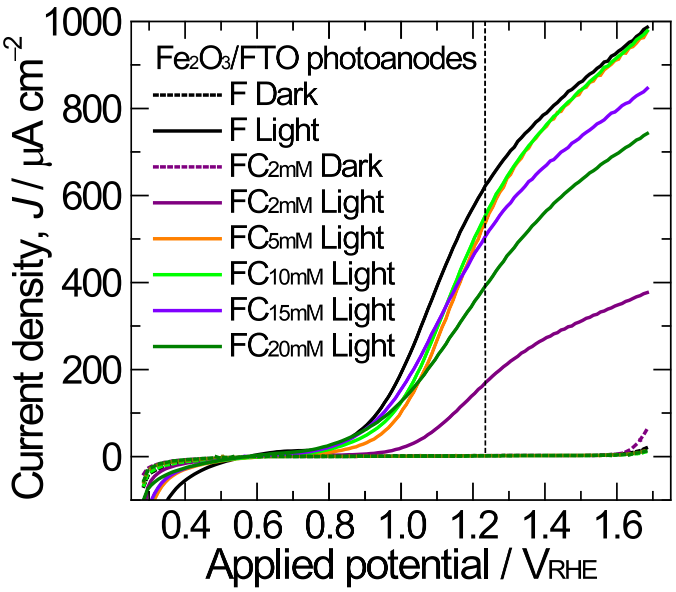

Figure S6. *J*–*V* characteristics of Fe_2O_3 photoanodes fabricated with and without surfactant (CTAB concentrations: 2, 5, 10, 15, and 20 mM). Scan rate: 20 mV s^{-1}; Illumination: 1 sun; Electrolyte: 1 M NaOH.

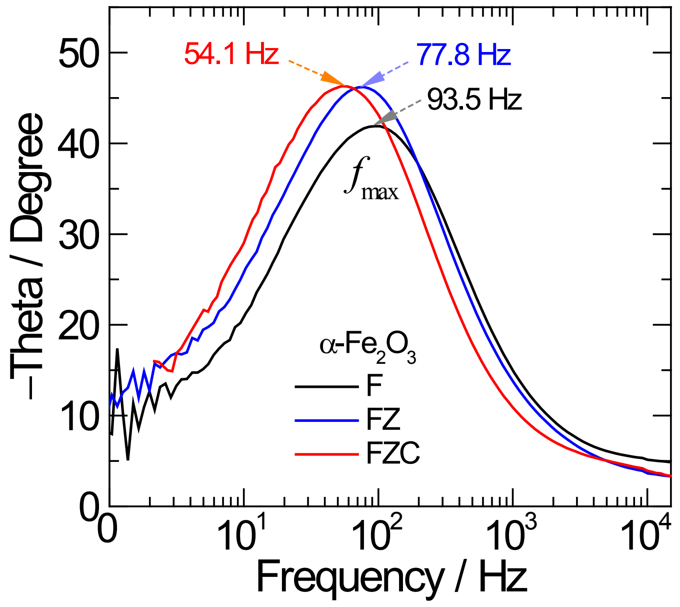

Figure S7. Bode phase plot of Fe_2O_3 photoanodes synthesized using ZrO_2 UL and surfactant (F, FZ, and FZC). The plots were obtained at a water splitting potential under 1 sun illumination in 1 M NaOH electrolyte.
